# In Memoriam—Temple S. Hoyne, A. M., M. D.

**Published:** 1899-03

**Authors:** 


### IN MEMORIAM—TEMPLE S. HOYNE, A. M., M. D.

Like a clap of thunder in a clear sky came the news of the death of this uncompromising disciple of Hahnemann. For years he had suffered in an indefinite sort of way, thinking there might be some organic lesion in first one part of the body and then some other more or less intimately associated portion of the body, but, by judicious living, succeeded in avoiding the sick-bed. Feeling in usually good health, he submitted to an exploratory incision into the bladder, Sunday, January 29th, which revealed nothing abnormal beyond an encysted calculcus, which was no longer an active source of irritation.

The recovery from the operation was marked with no untoward symptoms until the third day, when, by reason of a violent fit of coughing the stitches were torn out, followed by evidence of some profound disturbance within the abdomen. The moments in which hope was held out gradually grew farther and farther apart until the seal of death was written upon his brow. He died Friday morning, February 3d.

Temple Smith Hoyne, was born in Chicago, October 16th, 1841; he was the oldest son of the Hon. Thomas Hoyne, a lawyer and ex-Mayor of the city of Chicago, and was named from his grandfather, J. T. Temple, M. D., of St. Louis, and one of the pioneers of Homœopathy in the West, and his uncle, Dr. D. S. Smith, another staunch disciple of the then despised Hahnemann. It was the desire of his father that he be prepared to succeed him in his legal pursuits, but the influence of his mother, combined with that of his grandfather and uncle, led him to the selection of the healing art as his future avocation.

Graduating with high honors in the Chicago University, Class of '62, he entered Hahnemann, of Chicago, in the fall of the same year; but believing surgery offered greater inducements to the young man (it was in the midst of the civil war) than medicine, he went to New York in the spring of 1863 and entered the office of Dr. Frank H. Hamilton, the eminent surgeon, and graduated from Bellevue Hospital College in 1865.

During the campaign of 1864 he saw active service at the front, assisting Dr. Hamilton.

In the summer of 1865 he returned to Chicago, and, having surgery in view, was elected to the Chair of Pathology and prosector for the Professor of Anatomy; but a combination of circumstances dampened his surgical ardor, and he turned the full power of his strong, well-balanced mind into the domain of medicine and materia medica in particular. It was through his instrumentality that the valuable proving of *Carbolic-acid* was given to the profession.

Early in his professional life he established the habit of systematic reading and classified the same in an *Index Rerum* so that everything of value could be found at a moment's notice. Out of this grew his *Clinical Therapeutics*, a valuable demonstration of the action of remedies employed in accord with the law of *similia similibus* for the healing of the sick. (At the time of his death he had in preparation MSS. for a third volume, which should be carried through to completion by some trusted friend of the deceased.)

In the course of time the value of his services became recognized by the Board of Trustees of Hahnemann, and he was elected both Registrar and Treasurer of that institution. While in this office it became important for him to have a complete and reliable list of homœopathic physicians. Out of this grew the *Medical Visitor*, a publication known to the majority of the profession.

During the period in which he held the department of Skin and Venereal diseases, he felt handicapped because of the limited literature suited to the needs of the medical student, and with his accustomed thoroughness he wrote a book on skin and venereal diseases, that serves as a valuable guide to the selection of the indicated remedy.

His professional career of thirty-five years has been marked by unswerving loyalty to the cause of Homœopathy, and his professional confréres showed their appreciation of his excellent worth by electing him to many places of trust, his last post of honor being that of Dean of Dunham Medical College and Hospital.

For years he was a prominent member of the Iroquois Club, but his greatest enjoyment was found in the quiet gathering of congenial friends about the social board.

In 1866 his life was united with that of Miss Vedden, and his unexpected departure is mourned by the wife and their only daughter, Mrs. Bruce.—*The Hahnemannian Advocate.*

